# miRTissue _*ce*_: extending miRTissue web service with the analysis of ceRNA-ceRNA interactions

**DOI:** 10.1186/s12859-020-3520-z

**Published:** 2020-09-16

**Authors:** Antonino Fiannaca, Laura La Paglia, Massimo La Rosa, Riccardo Rizzo, Alfonso Urso

**Affiliations:** CNR-ICAR, National Research Council of Italy, via Ugo La Malfa 153, Palermo, 90146 Italy

**Keywords:** ceRNA interaction, miRNA-target interaction, miRNA sponge, Tumour, TCGA

## Abstract

**Background:**

Non-coding RNAs include different classes of molecules with regulatory functions. The most studied are microRNAs (miRNAs) that act directly inhibiting mRNA expression or protein translation through the interaction with a miRNAs-response element. Other RNA molecules participate in the complex network of gene regulation. They behave as competitive endogenous RNA (ceRNA), acting as natural miRNA sponges to inhibit miRNA functions and modulate the expression of RNA messenger (mRNA). It became evident that understanding the ceRNA–miRNA–mRNA crosstalk would increase the functional information across the transcriptome, contributing to identify new potential biomarkers for translational medicine.

**Results:**

We present miRTissue _*ce*_, an improvement of our original miRTissue web service. By introducing a novel computational pipeline, miRTissue _*ce*_ provides an easy way to search for ceRNA interactions in several cancer tissue types. Moreover it extends the functionalities of previous miRTissue release about miRNA-target interaction in order to provide a complete insight about miRNA mediated regulation processes. miRTissue _*ce*_ is freely available at http://tblab.pa.icar.cnr.it/mirtissue.html.

**Conclusions:**

The study of ceRNA networks and its dynamics in cancer tissue could be applied in many fields of translational biology, as the investigation of new cancer biomarker, both diagnostic and prognostic, and also in the investigation of new therapeutic strategies of intervention. In this scenario, miRTissue _*ce*_ can offer a powerful instrument for the analysis and characterization of ceRNA-ceRNA interactions in different tissue types, representing a fundamental step in order to understand more complex regulation mechanisms.

## Background

The cross-talk between coding and non-coding RNA molecules represents a novel layer of gene regulation. MicroRNAs (miRNA) are defined as "master regulators" of gene expression, as they can influence gene expression of RNA messenger (mRNA) through the direct binding on their MRE (miRNA Responsive Element) sites [1]. Apart from mRNA, other non-coding RNA (ncRNA) molecules can bind miRNAs through MRE sites. They are called competitive endogenous RNA (ceRNA) or miRNA sponge because they compete with mRNA target for miRNA binding [2, 3]. To date, the following ncRNA molecules have been experimentally validated having a ceRNA behaviour: long non-coding RNAs (lncRNAs), pseudogenes, and circular RNAs (circRNA). Some other classes of ncRNA molecules that work as ceRNAs, such as the tRNA-derived small RNAs (tsRNA) that have been recently associated with gene expression regulation in cancer progression [4–6], have a few pieces of evidence not yet experimentally validated.

ncRNAs are defined as regulative molecules because they can interfere with miRNAs in gene regulation process. As a consequence, an ncRNA action can alter a specific miRNA-target interaction through miRNA sequestration. Indeed they seem to have a relevant function in different physiological and pathological conditions as the development of human cancers [7, 8].

**Fig. 1:**
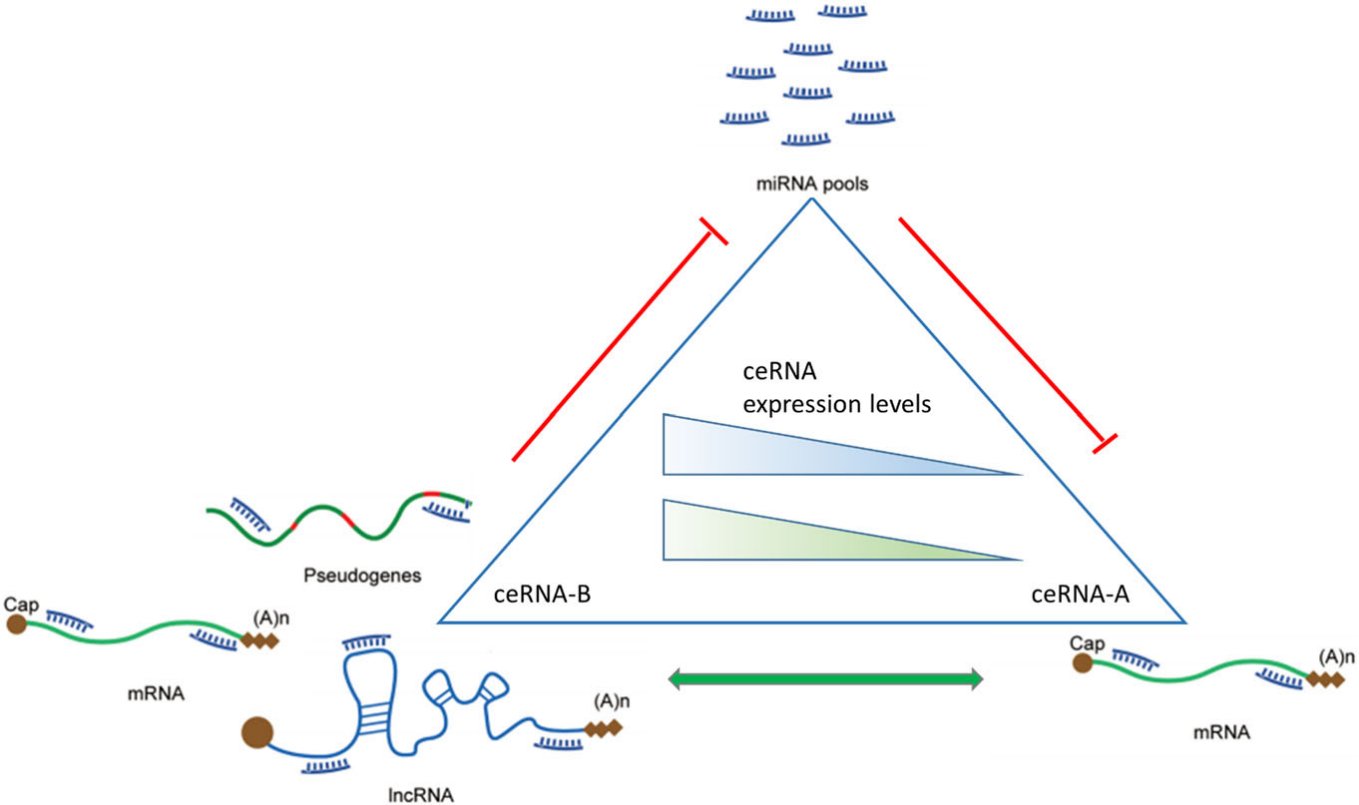
ceRNA interaction network. Figure shows ceRNA network interaction. The triangle represents the crosstalk of different ceRNA molecules. RNA molecules showed in each vertex of the triangle are different RNA molecules involved in ceRNA crosstalk. mRNA (ceRNA-A) interact with miRNAs trough MRE binding sites, and its expression is inhibited by miRNA linkage. miRNA can be also regulated himself by interacting with other ceRNA molecules (ceRNA-B) (lncRNAs, pseudogenes, mRNAs), by MRE site interaction with their seed sequence. ceRNA crosstalk is also influenced by the expression level of all RNA molecules that take part in the network. The functional balance of gene network in a specific cell or tissue is due to transcriptional levels of ceRNA-A and ceRNA-B molecules, and they behave like "competitors" for the same miRNA cluster

Salmena et al. [2] presented a unifying "ceRNA-hypothesis", in which different ncRNAs classes would actively talk to each other, through a "ceRNA language", composing a large-scale interaction network, and guiding their respective expression levels [9]. This molecular network is dependent on different features as concentration and subcellular distribution of the different RNA molecules considered, and also on the cellular type. Figure 1 shows a schematic overview of that process. All those features can have a different impact on general developmental and metabolic processes in multiple tissues, especially on transcription-related functions [10]. Applying this RNA regulative network in the context of cancer, considering a gene with tumor-suppressor function, a miRNA molecule targeting this mRNA could block its expression, acting as an oncogenic miRNA; in this case, a ceRNA molecule could compete with the tumor-suppressor for the same miRNA and restore the gene function. In the next part of this Section we discuss in detail all the ncRNA molecules with ceRNA function.

**Fig. 2:**
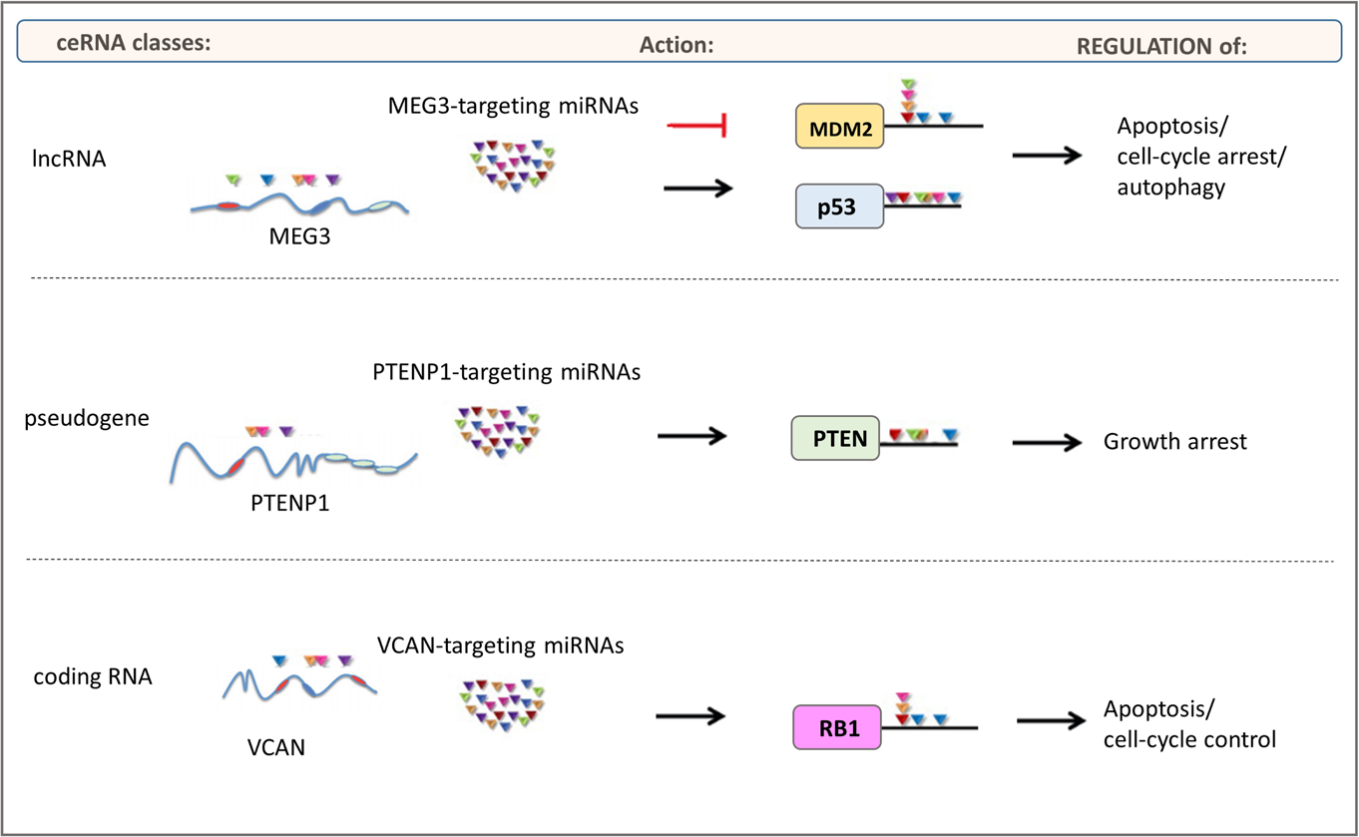
ceRNA classes. Figure shows some examples of different ceRNA molecules. lncRNA, pseudogenes and mRNA are represented. Figure shows also the mechanism of action of each ceRNA molecule, the regulation of cellular processes and validation study. For ceRNA classes of lncRNAs, two mechanisms of action are represented in figure: miRNAs acting on MDMD2, lead to its inhibition (red simbol); miRNAs acting on p53, lead to its activation (black arrow)

lncRNA are RNA molecules, more than 200 nucleotides long, involved in regulation of gene expression. In cancer context, several scientific works demonstrated that they are differentially regulated in tumor tissues compared with normal ones. One example is lncRNA Highly Up-regulated in Liver Cancer (HULC), highly over-expressed in hepatocellular carcinoma (HCC) [11]. MEG3 is another lncRNA which is involved in modulation of apoptosis and autophagy. It acts on p53 gene, enhancing its transcriptional activation, and on MDM2 gene, causing its downregulation. The final result of this molecular cascade is the block of cell-cycle and regulation of autophagy [12](Fig. 2).

Pseudogenes are segments of DNA highly homologous to their gene counterpart but "apparently" with no functional activity. Poliseno et al. hypothesized for the first time a ceRNA role for the phosphatase and tensin homolog (PTEN) pseudogene 1 (PTENP1) [9]: its sponge effect on PTEN gene leads to a decreased gene expression of PTEN and of other tumor suppressor genes. Moreover authors proved that cells presenting a PTENP1 overexpression show a growth inhibition, deducing a potential role of this pseudogene as tumor suppressor [9] (Fig. 2).

circular RNA (circRNA) is another class of non coding RNA molecules, called this way because of its covalently closed ring structure [13]. For instance, circTP63 is a circular RNA identified in differ cancer types [14].

More recently, there are evidences that mRNAs were associated with ceRNA functions. For example Lee et al. [15] showed that Versican (VCAN) 3’ UTR modulates retinoblatoma 1 (RB1) expression through specific miRNA interaction. It binds miR-199a and miR-144, upregulating the expression of this tumor suppressor gene (Fig. 2).

Considering the biological relevance of ceRNA molecules, in this paper we present an upgrade of the original miRTissue web service [16], called miRTissue _*ce*_, that stands for miRTissue "*c*eRNA *e*dition", integrating novel knowledge about ceRNA interactions. Built on the same platform and service architecture of its first release, miRTissue _*ce*_ now provides the possibility to search for ceRNA interaction networks in different tissues. Together with the basic functionalities of original miRTissue, that is the characterization of miRNA target interactions in different tissues and in different conditions (normal/tumour), miRTissue _*ce*_ offers a novel and complete insight about miRNA mediated regulation processes. Moreover, the study of intra-tissue (normal versus tumour) and inter-tissues (tumor A versus tumor B) correlations allows the user to better address its research of biological cancer markers in the field of precision medicine. Indeed, each tissue type has a proper biologic profile, with a proper expression profile for the same molecules under study, thus, a molecular marker identified for a specific tissue, could not be a marker for another tissue type.

miRTissue _*ce*_ was developed in order to improve the previous version of the tool, providing information on potential molecular markers in tumor biology, supporting the search for ceRNA interaction networks in many tissues. Its main impact is the research of potential endogenous molecules to use in precision medicine, in the field of prognosis, prediction, and cancer treatments.

The rest of the paper is organized as follows. In the next section, a set of similar bioinformatics services is reviewed. In “Materials and methods” section, we present the data sources, software packages and computational pipeline employed in our service. In “Results” section we describe miRTissue _*ce*_’s new features and new functionalities and then we introduce some case studies. In “Discussion” section, we point out the potential impact of our system and provide a comparison with other similar services. Finally “Conclusion” are drawn.

## Related works

In recent years, some bioinformatics services focused on the analysis of miRNA-mediated interaction networks. One of the first database collecting these interaction networks is miRTarBase [17]. miRTarBase collects only experimentally validated interactions obtained from published papers. Curators also collect information from other databases such as Human MicroRNA Disease Database (HMDD) [18], National Center for Biotechnology Information (NCBI) Entrez Gene [19] and NCBI Reference Sequence (RefSeq) [20], The Cancer Genome Atlas (TCGA) [21], Gene Expression Omnibus (GEO) [22], Kyoto Encyclopedia of Genes and Genomes (KEGG) [23] and Database of Annotation, Visualization and Integrated Discovery (DAVID) [24]. HMDD information are aimed to investigate the relationship between miRNA and human diseases. Among them NCBI RefSeq and Entrez Gene are used to obtain information about the target gene, while information about miRNA expression profiles are obtained from TCGA and Gene Expression Omnibus; DAVID and KEGG are used to obtain functional annotation of miRNAs.

ceRDB [25] is another database based on similar features. ceRDB provides putative ceRNA interactions starting from putative miRNA-mRNA interactions predicted by TargetScan [26]. The potential for miRNA-mRNA competition for each mRNA is ranked using the count of number of miRNA binding sites shared between the two RNA molecules (miRNA and mRNA). ceRDB considers as putative ceRNA only mRNA molecules.

ln*Ce*DB [27] collects from TargetScan the putative mRNA targets of human miRNAs, and from starBase [28] the targets predicted from available Argonaute (AGO) PAR-CLIP datasets, that is data obtained from Argonaute photoactivatable ribonucleoside-enhanced cross-linking and immunoprecipitation (PAR-CLIP) sequencing. Furthermore, the lncRNA targets of human miRNAs (up to GENCODE 11) are downloaded from miRcode database [29]. To find seed-matched target sites, miRNA targets on the rest of the GENCODE 19 lncRNAs are predicted by the developed prediction algorithm. These putative miRNA-lncRNA interactions are mapped to the AGO protein interacting regions within lncRNAs. The likelihood of a lncRNA-mRNA pair for actually being ceRNA is obtained by a two steps method. First, a ceRNA score is calculated from the ratio of the number of shared MREs between the pair with the total number of MREs of the individual candidate gene. Second, the hypergeometric test, using the number of shared miRNAs between the ceRNA pair against the number of miRNAs interacting with the individual RNAs, is used to calculate the *p*-value for each ceRNA pair. With the discovery of new ncRNA classes other databases collected and integrated information also on these new ncRNAs.

miRSponge [30] is a manually curated database providing an experimentally supported resource for miRNA sponges. The data are manually curated, and collected from PubMed literature, using about 1.200 published articles, and the database contains data on 599 miRNA-sponge interactions and 463 ceRNA relationships from 11 species. Database classes include endogenously generated molecules as coding genes, pseudogenes, long noncoding RNAs and circular RNAs, along with exogenously introduced molecules, including viral RNAs and artificial engineered sponges. Considering that lncRNA are not yet fully understood a wide spectrum of information is collected for each entry, such as pathway interactions from KEGG [23] and BioCarta [31], also integrated by information from TarBase [32], miR2Disease [33] and miRTarBase [17]. The functional information is obtained from Gene Expression Omnibus (GEO) [22] and supported by a "guilty-by-association" strategy.

LncACTdb 2.0 [34] is another database curating many ceRNA types such as circular RNAs and pseudogenes. It provides a comprehensive information on ceRNAs interaction networks in 23 species and 213 diseases/phenotypes, including 2663 experimentally supported, and manually curated, ceRNA interactions from more than 5000 published works of literature. LncACTdb also identifies and scores candidate lncRNA-associated ceRNA interactions across 33 cancer types from TCGA data, and providing illustration of survival, network and cancer hallmark information for ceRNAs. The system recalls the ceRNA interactions related to a lncRNA given as query, and then the user can access a set of tools to obtain more information. In particular new ceRNA interactions can be identified by integrating the expression profiles associated to a disease or a phenotype, and lncRNa functions can be studied through the pathways downloaded from repositories like KEGG, Biocarta and Reactome. Moreover new biomarkers can be evidenced by searching the survival time related to 33 kinds of cancer disease in TCGA database.

miRcode [29] is a searchable map of putative target sites across the whole set of sequences stored in GENCODE database of annotated transcriptome. The putative sites are obtained scanning the sequences for seed complementary fragments and considering the evolutionary conservation of the fragment. Evolutionary conservation is assessed by using multiple alignment of vertebrate sequences. Conservation level is evaluated checking the presence of the sequence in primates, non-primates mammals, and non-mammals vertebrates.

starBase v2.0 [28] is one of the first database collecting ceRNA interaction networks and it is also one of the most complete. starBase data sets are generated by 37 independent studies and the system can systematically identify the RNA–RNA, and protein–RNA interaction networks from 108 CLIP-Seq (PAR-CLIP, HITS-CLIP, iCLIP, CLASH). starBase v2.0 includes two web servers, miRFunction and ceRNAFunction, to predict the function of miRNAs and other ncRNAs from the miRNA-mediated regulatory networks. The database content includes, among others, the annotation and identification of miRNA-mRNA and miRNA-ncRNA interactions, and the annotation and identification of miRNA-mediated ceRNA regulatory networks. Regarding this content, the pipeline proposed in starBase combines CLIP-supported miRNA-mRNA, miRNA-lncRNA, miRNA-circRNA and miRNA-pseudogene interactions, then the hypergeometric test is implemented to predict ceRNA pairs among mRNAs, lncRNAs, circRNAs and pseudogenes. Recently, starBase v2.0 has been evolved and integrated into *ENCORI: The Encyclopedia of RNA Interactomes* (http://starbase.sysu.edu.cn). *ENCORI* is an open-source platform for studying the miRNA-ncRNA, miRNA-mRNA, ncRNA-mRNA, mRNA-mRNA, RBP-ncRNA, and RBP-mRNA interactions from CLIP-seq, degradome-seq and RNA-RNA interactome data.

## Materials and methods

In this Section, we describe data sources, computational tools and pipeline used in order to develop the new release of miRTissue web service. Moreover we provide a brief summary of the main functionalities of the earlier miRTissue release.

### RNA-target interactions

One of the data source of the presented pipeline are a set of RNA-target interactions, summarized in Table 1. To be more precise, the interactions are composed of a set of validated miRNA-target interactions (miRTarBase); a set of experimental validated ceRNA interactions (miRSponge and LncACTdb 2.0); a set of predicted miRNA-lncRNa and miRNA-pseudogenes interactions (miRcode); a set of experimentally validated miRNA-target interactions (miRTarBase).

**Table 1:** The data sources used in miRTIssue _*ce*_ and their data types

**Data source**	**Data type**	**Url**	**Ref.**
miRSponge	experimental validated ceRNA interactions	http://bio-bigdata.hrbmu.edu.cn/miRSponge/	[30]
LNCACTdb 2.0	experimental validated lncRNA-associated ceRNA interactions	http://www.bio-bigdata.net/LncACTdb/index.html	[34]
miRCode	Predicted miRNA-target interaction including lncRNA and pseudogene	http://www.mircode.org/index.php	[29]
miRTarBase	experimental validated miRNA-target interactions	http://mirtarbase.mbc.nctu.edu.tw/php/index.php	[64]
TCGA	Expression profiles of coding and non-coding RNA of different tissue types	https://www.cancer.gov/tcga	[65]

### RNA expression profiles

The other data sources of the computational pipeline are the expression profiles of several RNA molecules. Those data are extracted by the Cancer Genome Atlas project (TCGA, https://cancergenome.nih.gov/). TCGA repository was created in 2005 to improve the understanding of genetic bases of cancer disease, building a catalog of all genetic mutation responsible for cancer. In the following years the project demonstrated that a mutation atlas could be created for specific cancer types and that the public availability of the material enable researcher to make and validate research discoveries. Today TCGA stores data of more than 30 cancer types, including expression profiles of both coding and non-coding genes of tumor and healthy tissues In this work we consider expression profiles of the following bio types: protein coding, miRNA, pseudogenes, lncRNA. Among miRNA sponges, we will not discuss circRNA and tsRNA. The former because their expression values are not available in TCGA, the latter because there are only few pieces of evidence validating them as ceRNAs [4].

### miRTissue original release

miRTissue is a web service that allows to search for a tissue-specific characterization of miRNA-target interactions in human. Given a set of validated miRNA-target interactions provided by miRTarBase, the interaction type is computed according to a statistical correlation measure, using the global test [35], among the expression profiles of miRNAs, their mRNA targets and their corresponding proteins. Resulting interactions can be sorted by *p*-value or interaction types, and it is possible to organize the results for one or more tissue types, for example breast or colon. Given a single tissue, it is possible to check if the interaction type changes according to the tissue condition, that is normal or tumour. More details about the first release of miRTissue are available in [16].

### SPONGE software package

In the last years, many software packages have been released in order to compute *in silico* ceRNA interactions. For a review we recommend these papers [36–38]. For instance, authors in [38] proposed a new computational method, called ceRNA predIction Algorithm (CERNIA). CERNIA can be used to study the ceRNA competition among different tissue types, and different classes of genes. The ceRNAs prediction method is based on the DT-Hybrid recommendation algorithm [39, 40].

One of the most recent and most performing ceRNA predictor, according to its authors, is SPONGE (Sparse Partial correlation ON Gene Expression) [41]. SPONGE is an R software library that allows for large-scale inference of ceRNA interactions using a statistical correlation measure called multiple sensibility correlation (*mscor*). mscor needs an in-deep analysis because it is one of the main steps of miRTissue _*ce*_ processing pipeline. In particular, *mscor* is an extension of the basic sensitivity correlation, introduced by [42], that takes into account the effect of multiple miRNAs for the regulation of ceRNA interactions. It is defined as follows:

1 $$ mscor(g_{a},g_{b},M) = cor(g_{a},g_{b}) - pcor(g_{a},g_{b}|M)   $$

where *g*_*a*_ and *g*_*b*_ are the two genes involved in the ceRNA interaction, M is the set of shared miRNAs between *g*_*a*_ and *g*_*b*_,*cor*() is the Pearson correlation between expression profiles, *pcor*() is the partial correlation that estimates how two variables are correlated when they control additional variables. In this context, *mscor* gives an indication about the direct interaction, or less, between the two competing genes (*g*_*a*_ and *g*_*b*_) [42]. Indeed, a value of *mscor* close to zero means a direct interaction between the two observed variables, that is they are low sensitive to the presence of miRNAs. On the other hand, a value of *mscor* close to *cor*(*g*_*a*_,*g*_*b*_) means that there is a great contribution of the explanatory variables, that is the miRNAs, leading to an indirect correlation between *g*_*a*_ and *g*_*b*_, or, in other words, their interaction is miRNA-mediated. As explained in [34, 36], computational methods for inferring ceRNA interactions, based on statistical correlation, consider the following events: positive correlation between expression profiles of the two involved ceRNAs; negative correlation between the expression profiles of shared miRNA and the ceRNAs. Moreover, SPONGE method defines an *mscor* null distribution that allows to estimate an empirical *p*-value for *mscor*.

### miRTissue _*ce*_ computational pipeline

**Fig. 3:**
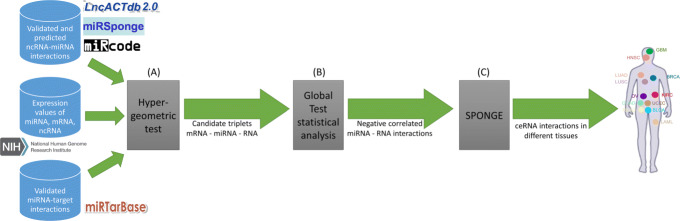
Computational pipeline for computing ceRNA interactions. The pipeline is based on the data extracted for different data sources (see Table 1). Using the hypethergeometric test (**a**) an initial set of putative ceRNA-A, miRNA, ceRNA-B triplets is found. Then we filtered only the couple miRNA-target having a negative correlation according to the globaltest (**b**). Finally we apply the SPONGE algorithm to the ceRNA interactions obtained in the previous steps (**c**). The resulting ceRNA interactions are computed for each cancer tissue

The new functionalities of miRTissue _*ce*_ service are provided by means of a three-step novel computational pipeline (Fig. 3) with regards to the one presented in [16]. The data sources are a set of RNA-interactions (see Table 1) and the expression profiles of considered RNA molecules taken from TCGA repository, as previously explained.

In order to compute a set of predicted ceRNA interactions, the first step (A) of our pipeline is to filter a statistical meaningful set of triplets in the form of ceRNA-A (coding gene) - ceRNA-B (RNA acting as sponge, including coding genes, pseudogenes and lncRNA) - list of putative miRNAs interacting with both ceRNA-A and ceRNA-B. As suggested in [36], this first step is done using the hypergeometric test. Given a couple of RNA, ceRNA-A and ceRNA-B, the hypergeometric test checks for the significance of the set of miRNAs interacting with both ceRNAs. The associated *p*-value to the test can be computed using the following formula:

2 $$ p = 1 - \sum^{x-1}_{i = 0} \frac{ \left(\begin{array}{c} K \\ i \\ \end{array}\right) \left(\begin{array}{c} N - K \\ O - i \\ \end{array}\right)} {\left(\begin{array}{c} N \\ O \\ \end{array}\right)}  $$

where *N* is the total number of available miRNAs, *K* is the number of miRNAs that interact with ceRNA-A, *O* is the number of miRNAs interacting with ceRNA-B, and *x* is the number of miRNAs shared by both ceRNA-A and ceRNA-B. The hypergeometric test is computed using the GDCRNATools R library [43]. The next filtering step (B) considers only the miRNA-target interactions that show a negative correlation between their expression values. That because, as explained in [16, 44], a miRNA and a target gene, in case they actually interacts, should have anti-correlated expression values. This is computed following the same approach as in miRTissue first release, that is using the globaltest statistical test [35, 44]. More details about the global test and its applications in bioinformatics can be found in [16, 35, 44]. The last step (C) of our computational pipeline is the application of the SPONGE method to the candidate ceRNA interactions obtained in the previous steps. The result of the pipeline is a table of ceRNA interactions in the form of ceRNA-A - ceRNA-B - number of shared miRNAs - list of shared miRNAs - mscor - *p*-value (Table 2).

**Table 2:** Sample table of the results obtained at the end of the computational pipeline, including the pair ceRNA-A/ceRNA-B, the number and the list of their shared miRNAs, *mscor* and its *p*-value (see Eq. 1)

**ceRNA-A**	**ceRNA-B**	**shared miRNAs**	**list of shared miRNAs**	**mscor**	***p*-value**
AASDHPPT	ERO1L	1	hsa-miR-98	0.0006	0.048
STOML1	RP11-290F5.2	2	hsa-miR-373; hsa-miR-372	0.0009	0.048
SESN1	RP11-290F5.2	2	hsa-miR-17; hsa-miR-519d	0.0115	0.039
ESR1	RP11-290F5.2	2	hsa-miR-373; hsa-miR-372	0.0007	0.049

## Results

In this section, we first present the new functionalities of miRTissue _*ce*_, then we discuss the "bioinformatics scenarios", previously presented in the earlier release of MirTissue web service, that now integrates the ceRNA interaction analysis: (1) ceRNA therapeutics analysis in cancer, (2) biomarker discovery in cancer, (3) ceRNA interaction network analysis in cancer.

### miRTissue _*ce*_ new functionalities

All the functionalities of miRTissue _*ce*_, including those of the previous release, are explained in detail in Additional file 1. In this Section, we briefly describe the new functionalities. miRTissue _*ce*_ offers two new use cases that can be selected from the home page.

In the first use case, called **ceRNA interaction analysis-compare among different tissues**, the user can input from a self completing list one or more mRNA name (ceRNA-A) and/or one or more ceRNA molecule name (ceRNA-B) and can select a list of different tissue. Then, for each tissue, it is possible to visualize the desired ceRNA pairs, if available, and the corresponding *p*-value. Moreover, by clicking on the *p*-value referred to a specific tissue, it is possible to switch to the visualization of the involved miRNA list in that tissue (Figure A3 of Additional file 1).

In the second use case, called **ceRNA interaction analysis-details for a specific tumor type**, the user can input from a self completing list one or more mRNA name (ceRNA-A) and/or one or more ceRNA molecule name (ceRNA-B). Then, after selecting one tissue type it is possible to obtain the related list of ceRNA interactions. Each pair is scored by a *p*-value and there is also a list of miRNAs which ceRNA-A and ceRNA-B compete for. By clicking on a miRNA in the list, it is possible to visualize all its mRNA targets for that tissue. The last column shows the number of other available tissues that exhibit that ceRNA pair (Figure A4 of Additional file 1).

Finally, the original miRNA-target use case, called **details for a specific tumor type** has been improved. Now, in fact, it is possible to realize if a miRNA-gene pair is involved in a ceRNA-ceRNA interaction (Figure A2 of Additional file 1).

### Bioinformatics scenario 1: ceRNA therapeutics analysis in cancer

**Fig. 4:**
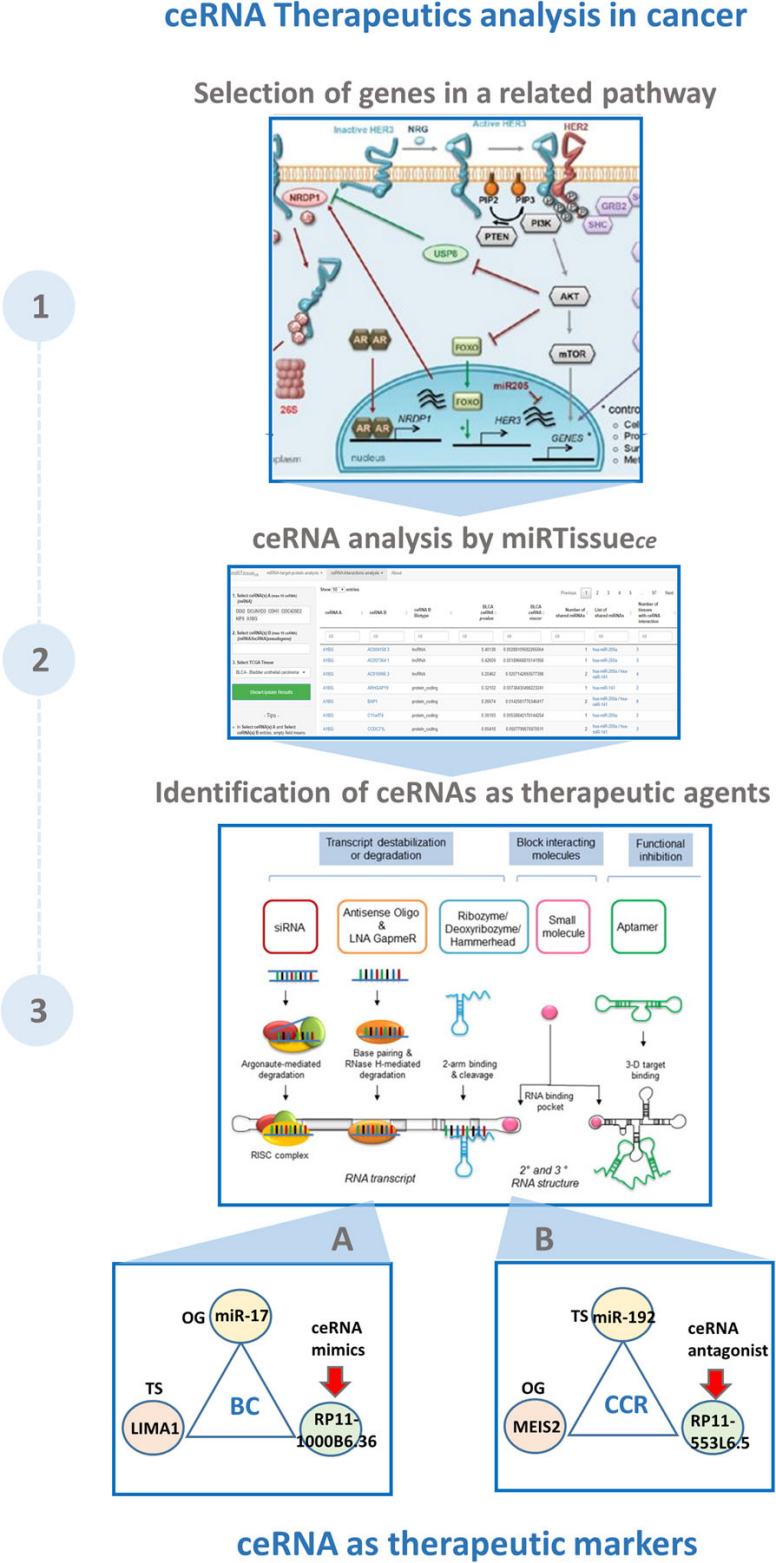
Bioinformatics scenarios. Bioinformatics scenarios: We present an example of common computational pipelines using miRTissue _*ce*_, in order to improve the investigation of current bioinformatics tasks. The upper part refers to "ceRNA Therapeutics analysis in cancer", the central part refers to "biomarker discovery in cancer", the lower part refers to "ceRNA interaction network analysis in cancer"

In last decade many studies focused their attention on the clinical use of synthetic ncRNA molecules for cancer treatments, as miRNA mimics or antagomirs, that mimic the functions of endogenous miRNAs, acting respectively on oncogenes or tumor-suppressor genes [45]. The identification of ncRNAs that are involved in cellular processes, contribute to oncogenesis, or tumor suppression, or act on miRNAs (well known regulators of gene expression), provides opportunities to develop novel therapeutics for cancer based on targeting lncRNA [46–48]. Several in silico molecules have been developed for this purpose, as small interfering RNAs (siRNAs), acting on specific ncRNAs [46], Natural Antisense Transcripts (NATs) [49], and AntiSense Oligonucleotide (ASO) [50]. Indeed, the development of this in silico molecules, can be an RNA-based drugs strategy for precision medicine, not only for treating cancer but also for modulating cancer treatment sensitivity. These molecules offer many advantages compared to other targeted therapies, such as fewer side effects and high specificity compared to other chemical drugs [51]. Moreover some cancer sub-types, as triple negative breast cancer (TNBC), do not have adequate therapies, because of the molecular characteristics of this tumor type, and ceRNA molecules could offer a great opportunity to individuate and intervene on this BC sub-type [51]. Taking into account this scenario, we designed miRTissue _*ce*_ to allow the researcher to investigate on ceRNA therapeutics analysis in cancer (Fig. 4). To address this issue, few steps are required: first, selection of genes of a specific pathway of interest; second, identification of RNA expression and ceRNA network interaction starting from the selected genes in a specific tumor tissue; third, identification of potential ceRNA molecules (mRNA, lncRNA or pseudogene) acting on oncogenes or tumor-suppressors. The second step can be achieved through correlation and statistical analysis offered by miRTissue _*ce*_ (Fig. 4). This scenario could be useful to investigate new synthetic molecules acting as ceRNA antagonists or agonists to use in cancer therapeutics.

**Fig. 5:**
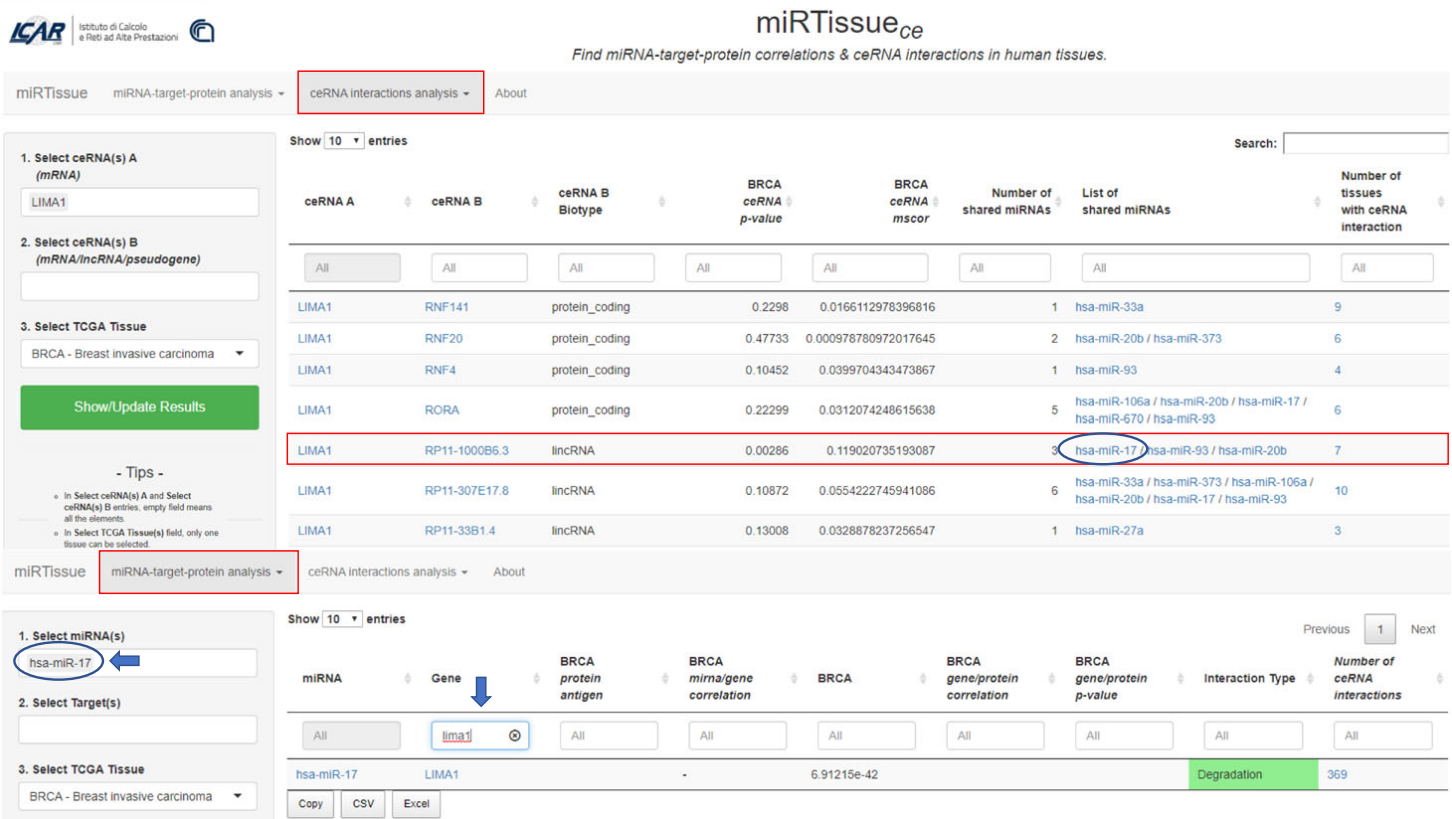
ceRNA network interaction analysis in BRCA tissue. ceRNA network interaction analysis is performed, through correlation and statistical analysis. In the example, filtering for Breast Cancer (BRCA) tissue, ceRNA network analysis evidenced an interaction between LIMA1 gene, has-miR-20b and has-miR-17 cluster and ncRNA RP11-1000B6.3. Both has-miR-20b and has-miR-17 are OncomiRs, and they act on LIMA1 tumor suppressor gene. Their interaction leads to LIMA1 mRNA degradation, thus decreasing its expression in breast cancer tissue (*p*-value <0.05). A ceRNA molecule (lncRNA, called RP11-1000B6.3) competing with LIMA1 for has-miR-20b and has-miR-17 cluster (*p*-value <0.05), is also evidenced by the web service

In order to better illustrate the use of miRTissue _*ce*_ in the analysis of ceRNA therapeutics application in cancer we report two specific examples:

A) filtering for Breast Cancer (BRCA) tissue, ceRNA network analysis evidenced an interaction between LIMA1 gene, has-miR-20b and has-miR-17 cluster and ncRNA RP11-1000B6.3 (Ensembl ID: ENSG00000261064). See Fig. 5 for interaction details. Both has-miR-20b and has-miR-17 are OncomiRs [52], acting on LIMA1 tumor suppressor gene [53]. Their interaction leads to LIMA1 degradation, thus decreasing its expression in breast cancer tissue (*p*-value <0.05). miRTissue _*ce*_ evidenced also RP11-1000B6.3 lncRNA, competing with LIMA1 for has-miR-20b and has-miR-17 cluster (*p*-value <0.05). A synthetic ncRNA molecule mimic RP11-1000B6.3 function could be used as therapeutic strategy to sequester those miRNAs and avoiding LIMA1 degradation.

**Fig. 6:**
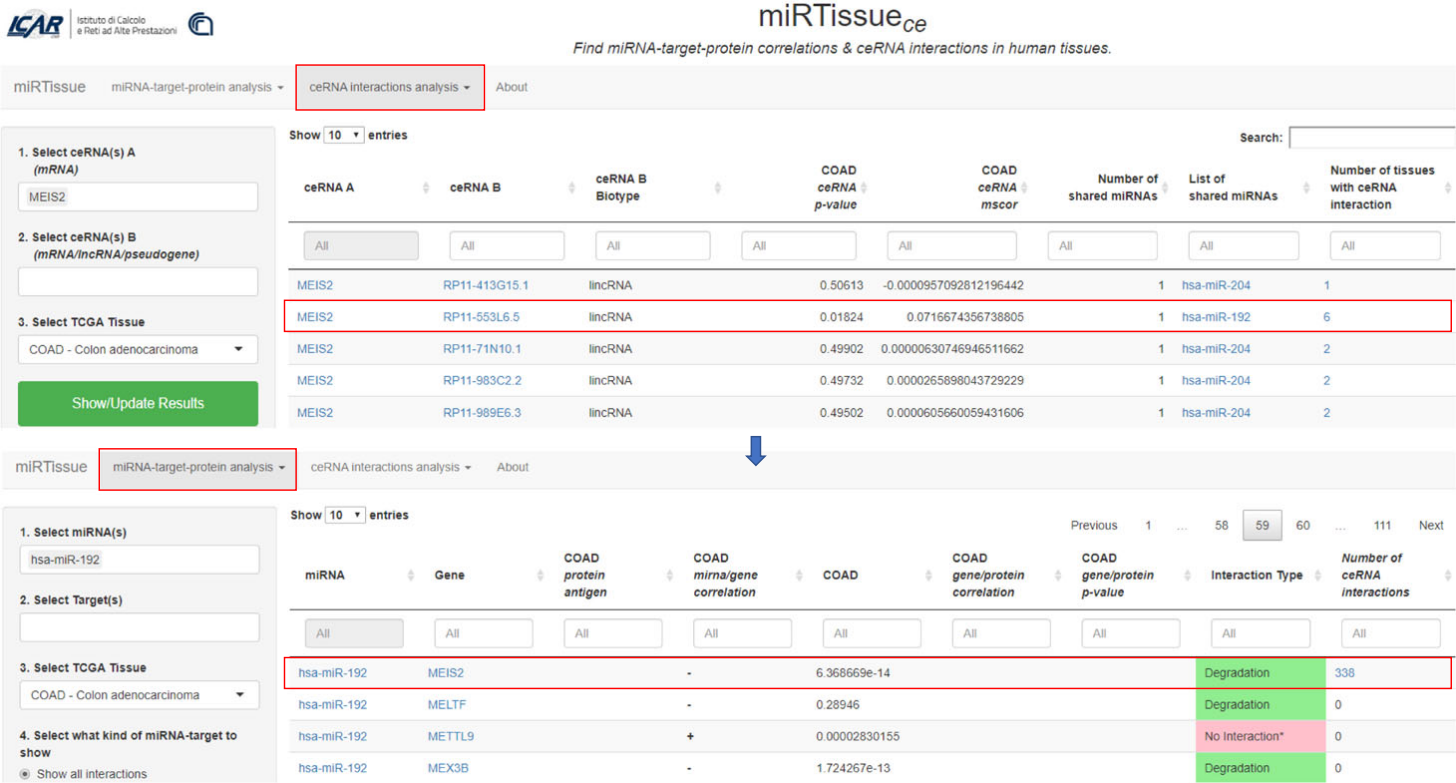
ceRNA network interaction analysis in COAD tissue. ceRNA network interaction analysis for Colorectal Adenocarcinoma (COAD) is shown in figure. MEIS2 gene-has-miR-192-lncRNA RP11-553l6.5 ceRNA triplette is evidenced by red rectangle

B) Another example, of ceRNA interaction evidenced by miRTissue _*ce*_ is in colorectal adenocarcinoma (COAD). See Fig. 6 for interaction details. MEIS2 oncogene interacts with has-miR-192, thus decreasing its expression. Experimental evidences support the role of MEIS2 as oncogene [54] in colon cancer tissue and the role of tumor suppressor of has-miR-192 [55]. They interact with lncRNA RP11-553L6.5. Synthetic ncRNA acting as antagonist could be used to inhibit RP11-553L6.5 function and restore the tumor suppressive miRNA function.

### Bioinformatics scenario 2: biomarker discovery in cancer

Cancer biomarkers are biological molecules widely used in different cancer stages, from diagnosis, disease staging or prediction, to clinical prognosis. Indeed they can indicate cancer evolution. There are biomarkers clearly related to disease initiation, or progression, and treatment effects [56]. ncRNAs could be considered good biomarkers for many reasons [57]: for example, their biological role is associated to gene expression regulation, and they are present in different body fluids, including whole blood, plasma, urine, saliva, and gastric juice [58, 59], making them easily detectable [60].

**Fig. 7:**
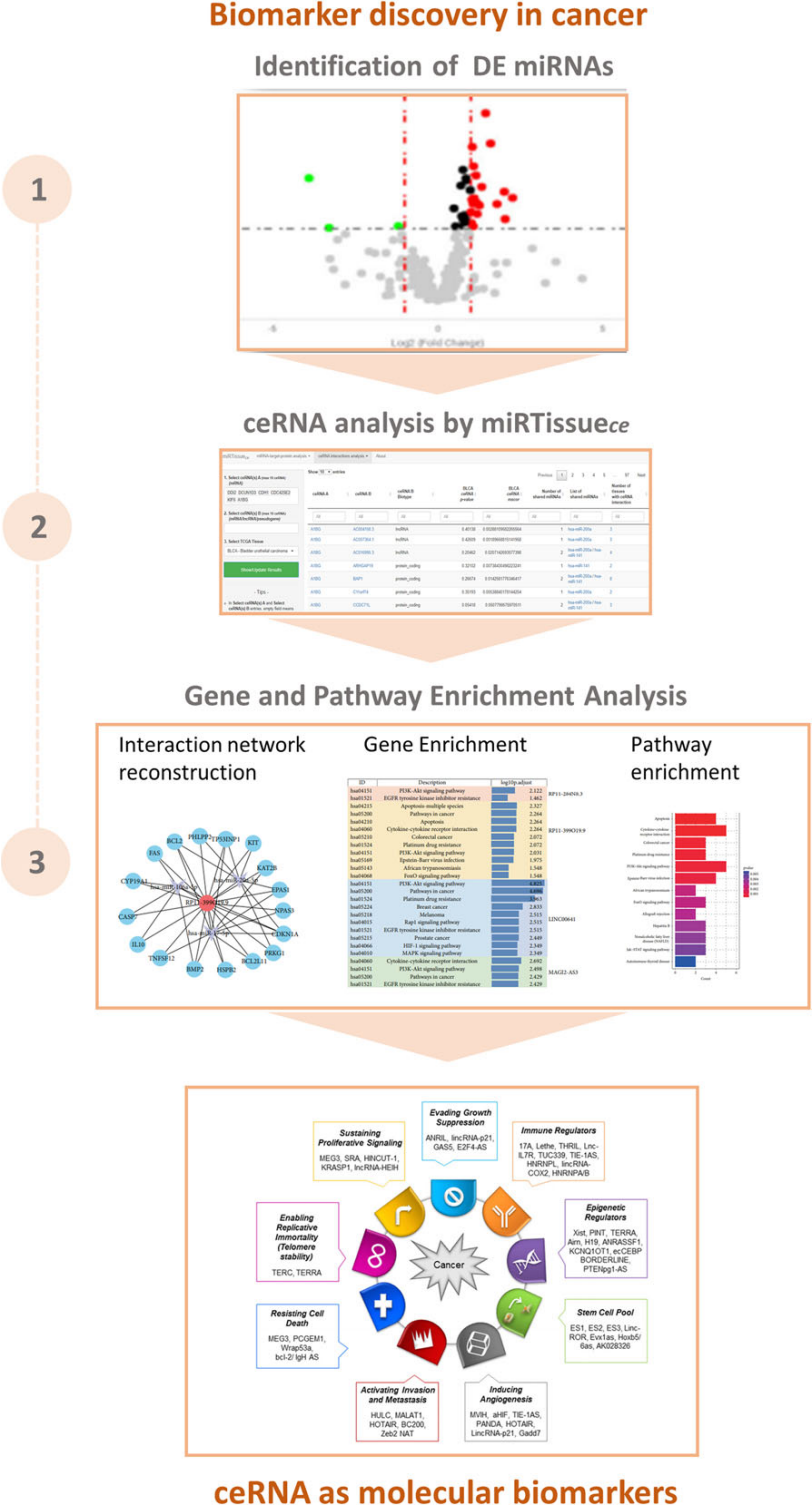
Bioinformatics scenarios. Bioinformatics scenarios: We present an example of common computational pipelines using miRTissue _*ce*_, in order to improve the investigation of current bioinformatics tasks. The upper part refers to "ceRNA Therapeutics analysis in cancer", the central part refers to "biomarker discovery in cancer", the lower part refers to "ceRNA interaction network analysis in cancer"

**Fig. 8:**
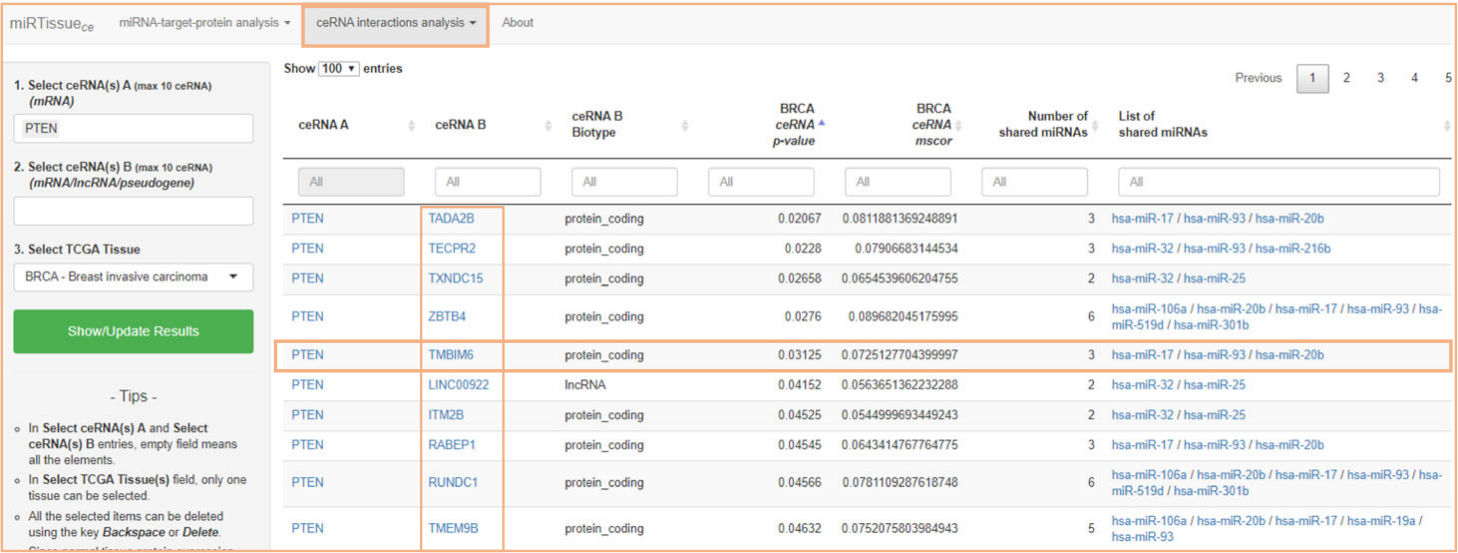
Case study on PTEN gene for Bioiformatics scenario 2: miRTissue _*ce*_ analysis. miRTissue _*ce*_ analysis for PTEN gene in Breast Cancer tissue, shows different ceRNA interactors

**Fig. 9:**
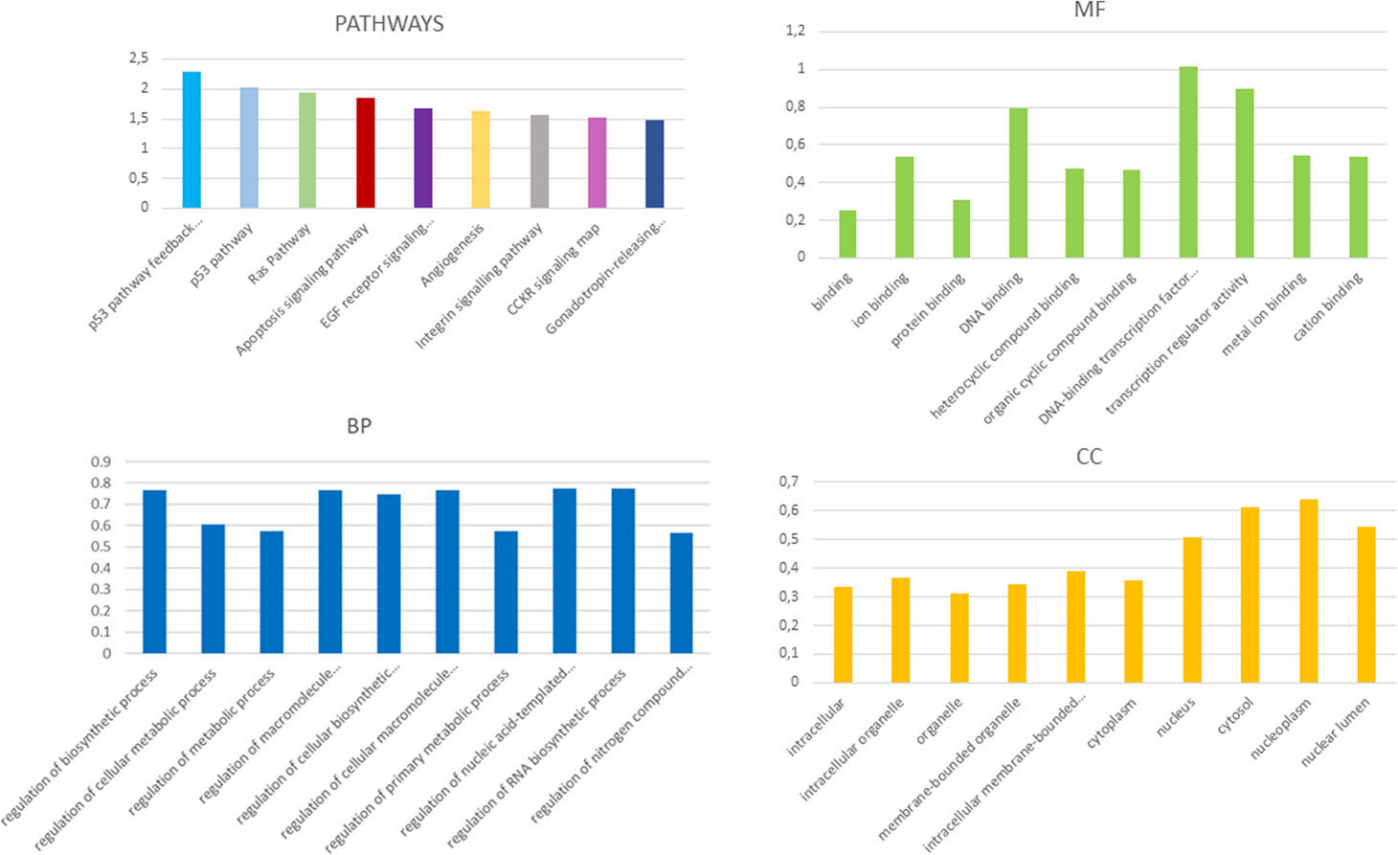
Case study on PTEN gene for Bioiformatics scenario 2: gene enrichment and pathway analysis of ceRNA competitors of PTEN gene. Both analysis were performed using PANTHER Classification System database (http://www.pantherdb.org/)): Gene Ontology (GO) Enrichment Analysis according to the three representative classes of GO (BP, MF, CC). Pathway analysis and GO classes showed in figure are filtered for *p*-value (<0.05) and FDR correction (<0.05) tests, according to Panther GO-slim analysis tool

To address the identification of new molecular biomarkers in cancer, few steps are required: first, identification of expression profiles of genes, miRNAs and other ncRNA molecules; second, reconstruction of ceRNA interaction network (miRNA-target, miRNA-ncRNA, ncRNA-target); third, functional characterization and pathway analysis of cancer specific ceRNA network. In this context, the use of miRTissue _*ce*_ would easily allow the researchers to get information on ceRNA interactions in the specific tissue under investigation, both considering expression values for all type of RNA molecules for each tissue, and evaluating the correlation between each couple of interacting molecules of the ceRNA network (Fig. 7). Indeed, this allows the user to know what are the exacts molecules expressed in a given tissue, as they could potentially interact with each other but not be expressed equally in all different tissues. Finally, functional characterization and pathway analysis would gain insight into the underlying biology of the ceRNA network and would determine the potential roles of lncRNAs that are aberrantly expressed in cancer. As result of this 3 step procedure, new potential ncRNA biomarkers could be evidenced. In order to better illustrate the use of miRTissue _*ce*_ in the analysis of biomarker discovery in cancer, we report a specific example: First of all, filtering for Breast Cancer (BRCA) tissue and for PTEN tumor suppressor gene, ceRNA network analysis evidenced an interaction between PTEN, many miRNAs, and ceRNA interactors (mRNAs, lncRNAs and pseudogenes). See Figs. 8 and 9 for interaction details. Figure 9 shows just the top 10 enriched categories, ranked for Fold Enrichment values. The full list of enriched genes is available as supplementary material (Additional file 2). We downloaded all ceRNAs interacting with PTEN gene and then we analyzed them through gene enrichment and pathway analysis. Gene enrichment evidenced an over-representation of the different Biological Processes (BP), Molecular Functions (MF) and cellular Component (CC) linked to regulation of the cellular bio-synthetic process, regulation of signal transduction, apoptotic process, Ras protein signal transduction, and cell death. The pathway analysis also showed enriched terms as p53 protein pathway, RAS protein pathway and apoptosis signalling. Indeed PTEN is a tumor suppressor gene which, in breast cancer cells, is involved in suppression of cell growth by phosphatase activity-dependent G1 arrest followed by cell death [61]. Moreover, a loss of PTEN expression is associated with poor outcome in this tumor type [62, 63]. Some of ceRNAs, as TMBIM6, are linked to pathways previously cited, and they interact with oncogenic miRNAs (hsa-miR-17/has-miR-20b) that regulate these biological processes. This analysis can, thus, evidence ceRNA biomarkers able to bind and block the action of oncogenic miRNA on tumor suppressor genes as PTEN in specific molecular networks (Figs. 8 and 9).

### Bioinformatics scenario 3: ceRNA interaction network analysis in cancer

**Fig. 10:**
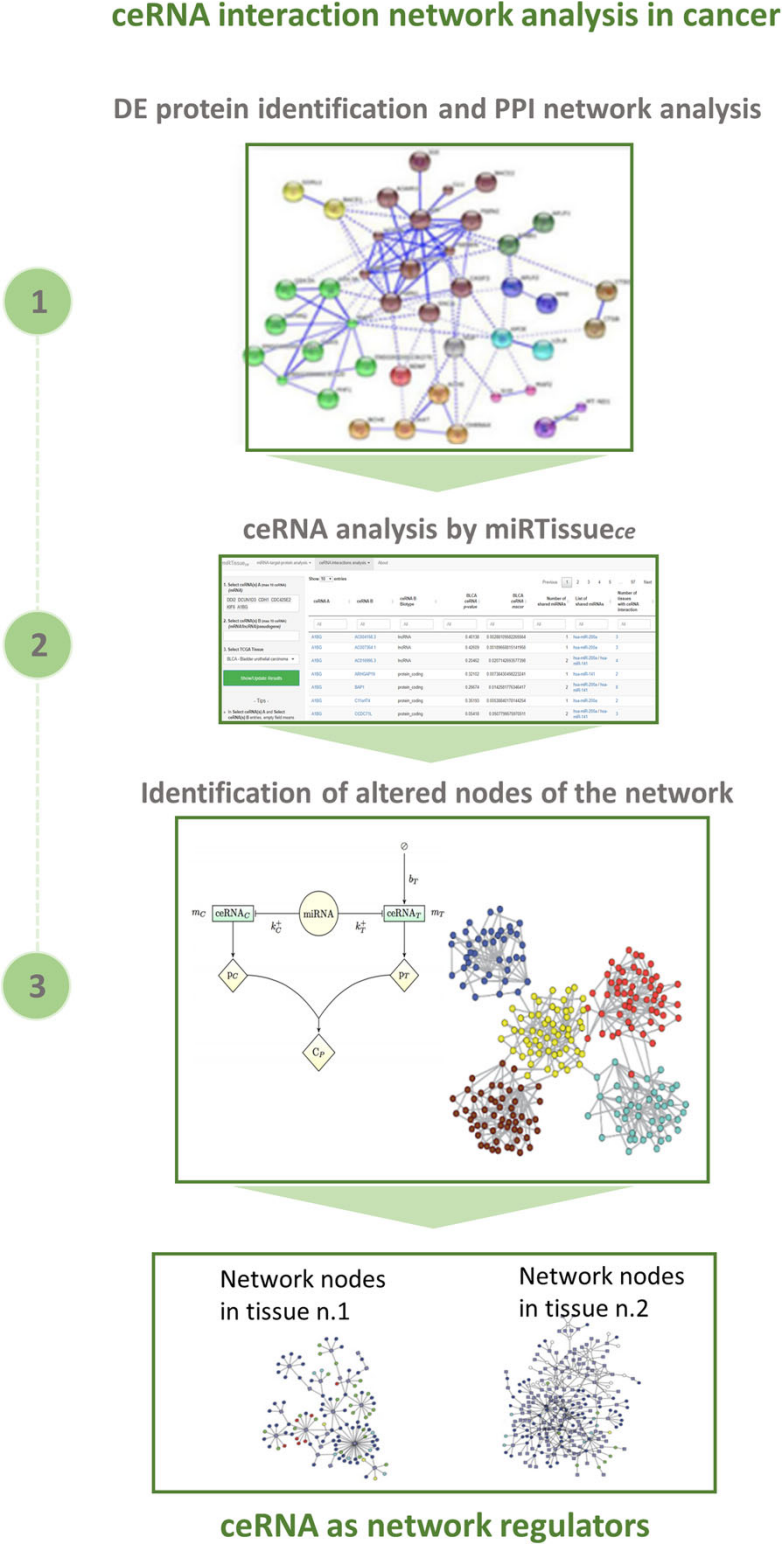
Bioinformatics scenarios. Bioinformatics scenarios: We present an example of common computational pipelines using miRTissue _*ce*_, in order to improve the investigation of current bioinformatics tasks. The upper part refers to "ceRNA Therapeutics analysis in cancer", the central part refers to "biomarker discovery in cancer", the lower part refers to "ceRNA interaction network analysis in cancer"

According to recent discoveries, mRNA can be considered a ceRNA molecule [15]. A mRNA-mRNA pair can, indeed, compete for the same miRNA or for the same miRNA cluster. Considering two different tissue conditions, the alteration of mRNA-mRNA interaction, translating it into protein-protein interaction (PPI) network, would potentially lead to modification of biological networks and cellular pathway dynamics. The impact of ceRNA network analysis in clinical research, and consequently the use of miRTissue _*ce*_ is linked to the deregulation and complexity of molecular interactions rather than a single molecule in complex diseases as is cancer. Through miRTissue _*ce*_ it is possible to evidence and study changes in the dynamic of the network in two different tissue types, by using these few steps: first, selection of differentially expressed (DE) genes of a specific pathway in two different tissue types (for instance normal and cancer tissue for the same tissue); second, reconstruction of ceRNA interaction network in the two conditions; and third, comparison of network modules and investigation of pathway dynamics (Fig. 10). miRTissue _*ce*_ allows to evidence and compare RNA interactions and consequently PPI network alterations.

## Discussion

Tumor biology is a complex scenario to investigate, due to its inter and intra-individual variability related to tumor tissue composition, in terms of both the wide numbers of molecules belonging to each tissue, and of quantitative expression. Those features can significantly modify pathway networks and the biological behaviour of a given tumor. In the context of precision medicine, in fact, each cancer patient has a unique patho-physiologic profile, strictly dependent on its molecular and genetic profiles. The knowledge of a cancer patient’s transcriptome can offer a comprehensive view of molecular patterns linked to cancer and can allow identifying biological molecules used as risk, diagnostic or prognostic markers as well as therapeutic targets.

miRTissue _*ce*_ was developed in order to provide information on ceRNA interaction networks in many tissues, and thus to address clinical research studies focusing on single patient molecular profile. It has features and functionalities that make it different with regards to other bioinformatics databases that provides similar information. Table 3 reports a comparison of miRTissue _*ce*_ features with other ceRNA databases. In particular, we identified the following seven main features:

- ceRNA interaction data source: the data repositories used to find ceRNA interactions;
- ceRNA classes: the considered bio-types of the molecules involved in ceRNA interactions;
- ceRNA network prediction algorithm: the algorithm, or computational pipelines, used to infer ceRNA networks;
- TCGA expression profiles for ceRNA interactions: whether or not TCGA expression profiles of ceRNA molecules are used;
- ceRNA interaction score *p*-value: whether or not a *p*-value over the score of a ceRNA interaction is used;
- multiple ceRNA analysis: whether or not it is possible to carry on analysis considering more than one ceRNA at a time;
- multiple tissue selection: whether or not it is possible to carry on analysis considering more than a tissue at a time.

**Table 3:** Comparison among the proposed miRTissue _*ce*_ database and other 7 ceRNA interactions databases, in terms of their main features

**Features**	**miRTissue**_***ce***_	**miRTarBase**	**ceRDB**	**lnCeDB**	**miRSponge**	**LncACTdb**	**miRcode**	**starBase v2.0**
**ceRNA interaction data sources**	miRTarBase	HMDD	TargetScan	TargetScan	TarBase	miRTarBase	TargetScan	miRBase
	miRCode	NCBI		StarBase	miRTarBase	miRanda		refSeq
	LncACTdb 2.0	RefSeq		miRCode	miRanda	TarBase		Ensembl database
	miRSponge	miRBase			miRecord			TargetScan
		miRanda						Pictar2, miRanda
		TargetScan						PITA, RNA22
**ceRNA classes**	miRNA-mRNA	miRNA-mRNA	miRNA-mRNA	miRNA-mRNA	miRNA-mRNA	miRNA-mRNA	miRNA-mRNA	miRNA-mRNA
	miRNA-lncRNA			miRNA-lncRNA	miRNA-lncRNA	miRNA-lncRNA	miRNA-lncRNA	miRNA-lncRNA
	miRNA-Pseudogenes				miRNA-Pseudogenes	miRNA-Pseudogenes	miRNA-Pseudogenes	miRNA-Pseudogenes
					miRNA-circRNA	miRNA-circRNA		miRNA-circRNA
								RBP-ncRNA
								RBP-mRNA
**ceRNA network**	hypergeometric test	N/A	N/A	hypergeometric test	hypergeometric test	hypergeometric test	N/A	hypergeometric test
**prediction algorithm**	+					+		
	global test					Pearson correlation		
	+							
	SPONGE							
**TCGA expression profiles**	Yes	No	No	No	No	Yes	No	Yes
**for ceRNA interactions**								
***p*-value on**	Yes	No	No	No	No	No	No	No
**ceRNA interaction score**								
**Multiple ceRNA analysis**	Yes	No	No	No	No	No	No	No
**Multiple tissue selection**	Yes	No	No	No	No	No	No	Yes
**References**	N/A	[17]	[25]	[27]	[30]	[34]	[29]	[28]

miRTissue _*ce*_, through the SPONGE algorithm, is the only system that provides a *p*-value related to a score, the *mscor* in this case, associated to a ceRNA interaction. Other systems that implement hypergeometric test, like for instance starBase v2.0, just provides a simple *p*-value associated to a candidate triplet ceRNA-A/ceRNA-B/miRNA, but there is not a score associated to that triplet, nor a corresponding *p*-value. One of the main characteristics of miRTissue _*ce*_ tool is the possibility to analyse many ceRNA triplets (ceRNA-A/miRNA/ceRNA-B) at the same time. Moreover the tool allows to analyse many tissues at the same time, giving the possibility to compare different tissues for the same ceRNA triplet under investigation. Other databases, such as ceRDB or miRSponge, give the user the possibility to analyse just one ceRNA at time, showing the miRNA cluster that interacts with the RNA target, and a list of putative ceRNAs. Moreover, miRTissue _*ce*_ is one of the few services that allows the user to rank ceRNA interactions by using a *p*-value score.

ceRNA interactions are all extracted from TCGA repository, this implies that ceRNA interaction analysis is homogeneous. Finally miRTissue _*ce*_, with respect to the most complete databases such as LncACTdb and ENCORI, implements in its pipeline the SPONGE algorithm that, at the best of our knowledge, is the most performing tools in order to compute ceRNA interactions.

## Conclusion

We presented miRTissue _*ce*_, a web service that represents an improvement of our original miRTissue release, extending its focus on the characterization of miRNA-target interactions. miRTissue _*ce*_, in fact, integrates data about RNA-target interactions and their expression profiles in order to provide, for different tissue types, a set of ceRNA-ceRNA interactions. RNA molecules acting as ceRNA, indeed, are fundamental elements in the cross-talk mechanism that involves gene expression regulation. The analysis of ceRNA networks in cancer tissues enables to widen clinical research in field such as biomarker discoveries and therapeutic strategies, as explained in the bioinformatics scenarios. With regards to existing similar bioinformatics services, miRTissue _*ce*_ implements a computation pipeline based on the state-of-the-art algorithm for inferring ceRNA-ceRNA interactions and it provides an easy way to search for ceRNA interactions in several cancer tissue types. Together with the functionalities of the previous release, miRTissue _*ce*_ offers a complete insight about miRNA mediated regulation processes.

## Supplementary information

**Additional file 1** Help pages for miRTissue _*ce*_ main functionalities.

**Additional file 2** Full list of enriched genes according to BP, MF, CC categories, and pathway analysis.

## Data Availability

miRTissue _*ce*_ is freely available at http://tblab.pa.icar.cnr.it/mirtissue.html.

## Abbreviations

AGO: Argonaute
ASO: AntiSense oligonucleotide
BC: Breast cancer
BP: Biological process
BRCA: Breast cancer
CC: Cellular Component
ceRDB: Vompeting endogenous RNA database
ceRNA: Competitive endogenous RNA
CERNIA: ceRNA predIction Algorithm
circRNA: Circular RNA
CLASH: Cross-linking, ligation and sequencing of hybrids
COAD: Colorectal adenocarcinoma
DAVID: Database of annotation, visualization and integrated discovery
DE: Differentially expressed
ENCORI: The Encyclopedia of RNA interactomes
GEO: Gene expression omnibus
GO: Gene ontology
HCC: Hepatocellular carcinoma
HMDD: Human MicroRNA disease database
HULC: Highly up-regulated in liver cancer
KEGG: Kyoto encyclopedia of genes and genomes
lncRNA: Long non-coding RNA
miRNA: microRNA
MF: Molecular function
MRE: miRNA responsive element
mRNA: Messenger RNA
mscor: Multiple sensibility correlation
NAT: Natural antisense transcript
NCBI: National center for biotechnology information
ncRNA: Non-coding RNA
PAR-CLIP: Photoactivatable ribonucleoside-enhanced cross-linking and immunoprecipitation
PPI: Protein-protein interaction
PTEN Phosphatase and tensin; PTENP1: Phosphatase and tensin homolog pseudogene 1
RB1: Retinoblatoma 1
RefSeq: NCBI reference sequence
RNA: RiboNucleic acid
siRNA: Small interfering RNA
SPONGE: Sparse partial correlation on gene expression
TCGA: The cancer genome atlas
TNBC: Triple negative breast cancer
tRNA: Transfer RNA
tsRNA: tRNA-derived small RNA
UTR: Untranslated region
VCAN: Versican
