# Supplementary material for: miRTissue ce: extending miRTissue web service with the analysis of ceRNA-ceRNA interactions
Source: BMC Bioinformatics. 2020 Sep 16;21(Suppl 8):199. doi: 10.1186/s12859-020-3520-z (PMC7493844; doi:10.1186/s12859-020-3520-z)
Supplement: Supplementary file 1 — Additional file 1 Help pages for miRTissue ce main functionalities. [file 12859_2020_3520_MOESM1_ESM.pdf]

Use case 1: miRNA-target-protein analysis

a) Compare Normal/Tumor miRNA-target interactions:

1. User can select one or more miRNA
2. User can select one or more gene Target
3. User can select one or more TCGA tissue
4. User can select Normal, Tumor or Normal & Tumor

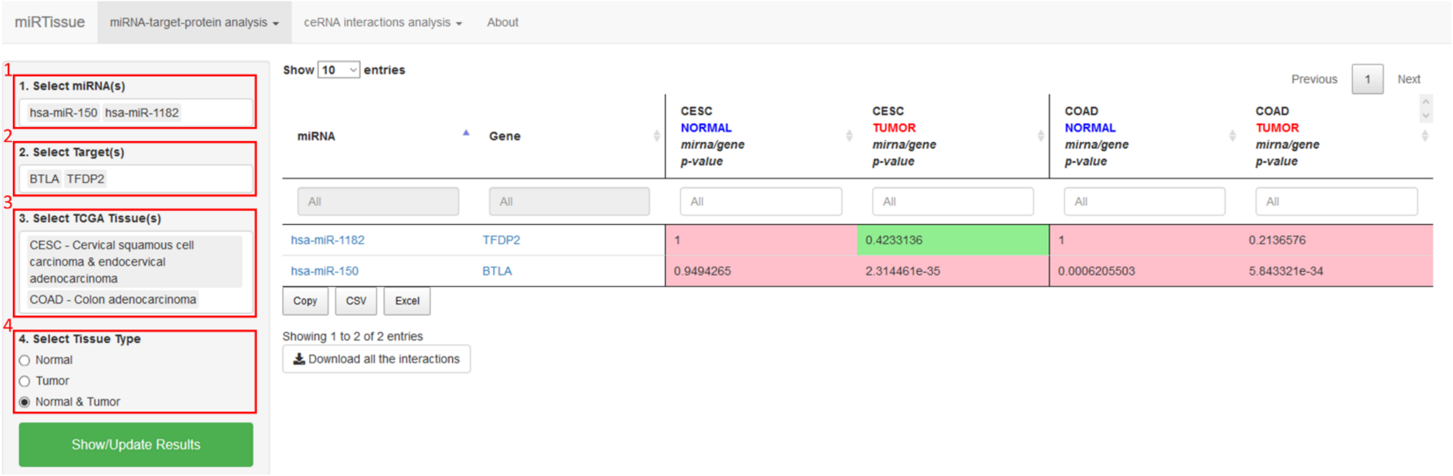

Figure 1

b) Details for a specific tumor type:

1. User can select one or more miRNA
2. User can select one or more gene Target
3. User can select only one TCGA tissue
4. User can select to show all interaction or only interactions where protein expression value is given
5. User can click on the value in the last column (number of ceRNA competitors) in order to visualize all ceRNA interactions that involve the selected miRNA and gene. (see use case 2a)

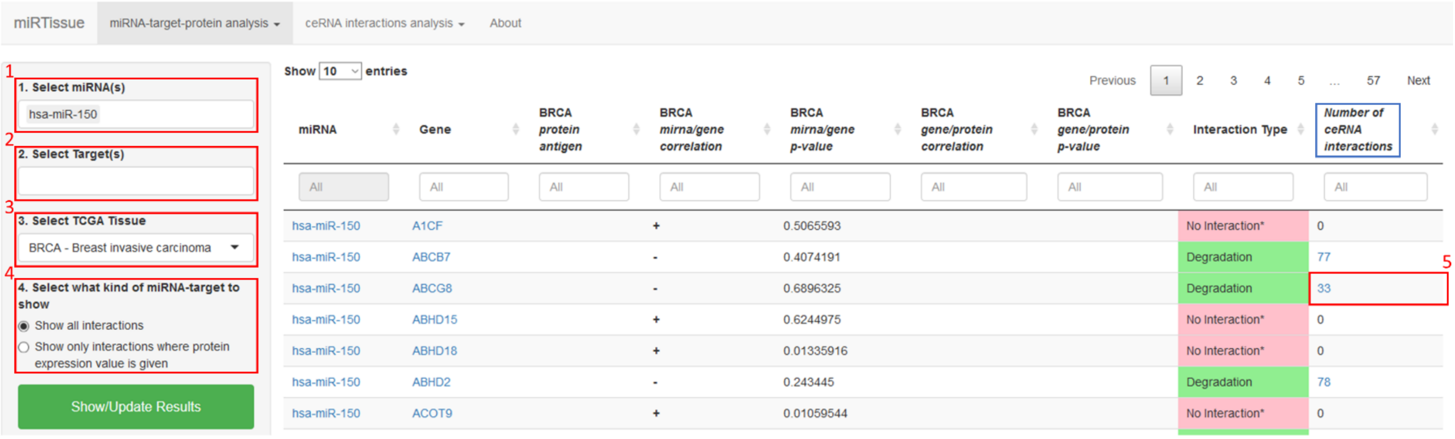

Figure 2

Use case 2: ceRNA interactions analysis

a) Compare among different tissues:
   1. User can select one or more ceRNA (mRNA)
   2. User can select one or more ceRNA (mRNA, lncRNA, pseudogene)
   3. User can select one or more TCGA tissue
   4. User can click on the p-value of a ceRNA interaction in order to visualize the list of shared miRNA (see use case 2b)

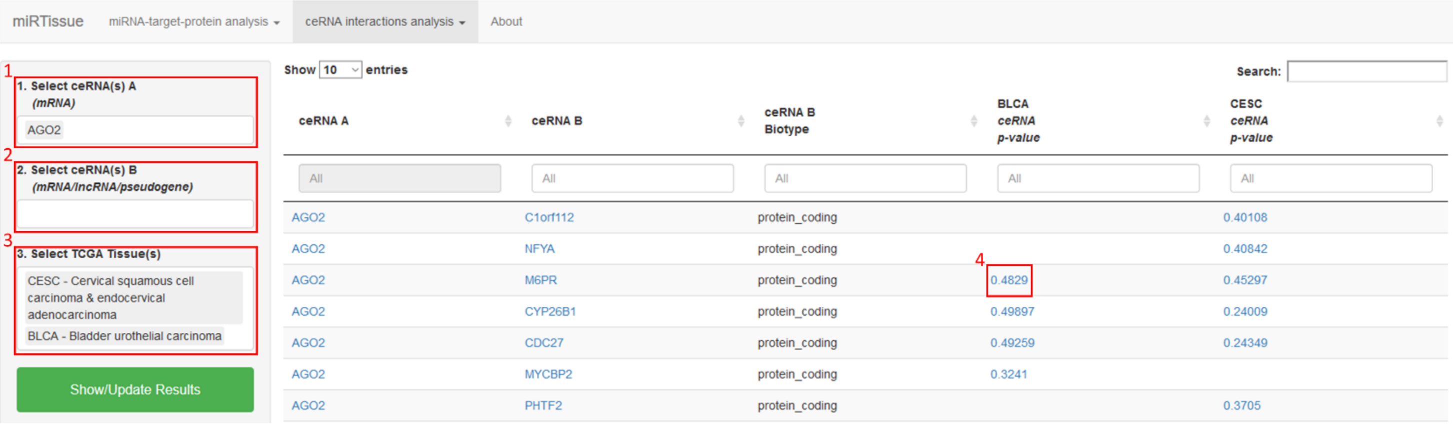

Figure 3

b) Details for a specific tumor type
   1. User can select one or more ceRNA (mRNA)
   2. User can select one or more ceRNA (mRNA, lncRNA, pseudogene)
   3. User can select only one TCGA tissue
   4. Use can click on the value of last column ("number of tissue having this ceRNA") in order to visualize the same ceRNA interaction in other tissues (see use case 2a)
   5. User can click on a miRNA of the "list of miRNA" in order to visualize all the miRNA target and interaction type for the selected miRNA (see use case 1b)

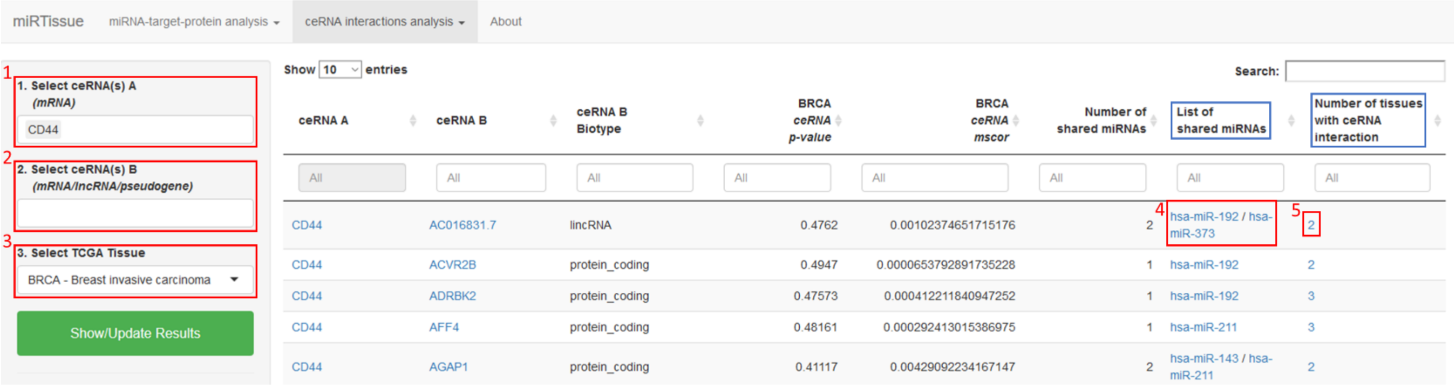

Figure 4
